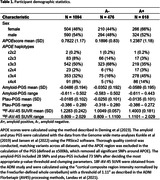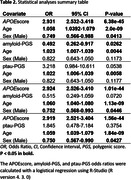# Alzheimer’s disease APOE neuropathology‐based score, amyloid, and ptau polygenic scores' association with amyloid PET positivity

**DOI:** 10.1002/alz.093681

**Published:** 2025-01-09

**Authors:** Yazan Hammad, Yuetiva Deming, Jacob Morse, Ethan Grover, Barbara B. Bendlin, Tobey J. Betthauser

**Affiliations:** ^1^ Wisconsin Alzheimer’s Disease Research Center, University of Wisconsin School of Medicine and Public Health, Madison, WI USA

## Abstract

**Background:**

APOE is the greatest genetic risk factor for AD, however, other smaller genetic effects are often ignored. In this work, endophenotype‐informed polygenic scores (PGS) that exclude the APOE region were tested along with a separate, previously published, APOE neuropathology‐based score (APOEscore). The APOEscore serves as a more nuanced quantification of APOE genetic risk that considers the effects of the different haplotypes. PGS and APOEscore were compared to amyloid positivity, determined via PET imaging, which is used as a measure for AD risk and progression.

**Methods:**

Alzheimer’s Disease Neuroimaging Initiative (ADNI) participants with genetic data and Florbetapir (18F‐AV‐45) amyloid PET summary SUVR (whole cerebellum reference region) values were included in analyses. Amyloid positivity (A+) was defined as SUVR > 1.11. PGS were calculated using the weights from genome‐wide association studies (GWAS) of CSF Aß42 (amyloid‐PGS) or ptau181 (ptau‐PGS), after excluding the APOE region (±500kb). APOE effects were accounted for using the APOEscore that was calculated following Deming et al (2023).

**Results:**

1094 participants (618 A+) were included in the analyses (Table 1). The APOEscore was significantly associated with A+ after accounting for sex and age at scan (OR = 2.931, P = 6.38e‐45; Table 2). The amyloid‐PGS was also significantly associated with A+ (OR = 0.492, P = 0.026), whereas the ptau‐PGS did not quite reach significance (OR = 3.218, P = 0.054). After adding the APOEscore, the effect of amyloid‐PGS on A+ remained but did not quite reach significance (OR = 0.515, P = 0.072) and ptau‐PGS was not significant (OR = 1.845, P = 0.375).

**Conclusion:**

These results validate the utility of the APOEscore with PET amyloid as well as the potential value of including non‐APOE genes in quantifying the genetic risk for amyloid accumulation and AD. The significant association between amyloid‐PGS and A+, and near significance after adding the APOEscore, demonstrates the existence of additional genetic effects outside of APOE that have an impact on amyloid accumulation. While the ptau‐PGS did not reach significance, possibly due to sample size, the OR (3.218) was greater in magnitude than the amyloid‐PGS (1/OR = 2.033). Future work will explore these relationships in other cohorts and with other PET AD biomarkers.